# The Role of Associative Learning in Current Paradigm Shifts in Eating Disorder Research and Clinical Practice

**DOI:** 10.3390/bs12110451

**Published:** 2022-11-14

**Authors:** David Garcia-Burgos

**Affiliations:** Department of Psychobiology, Institute of Neurosciences, Biomedical Research Centre, University of Granada, 18016 Granada, Spain; davidgb@ugr.es

## 1. Introduction

This editorial is an introduction to the Special Issue “Psychopathological analysis and intervention for anorexia nervosa: using associative-learning mechanisms”. Although learning theory was once the theoretical framework of choice for behavioural scientists interested in mental disorders, in recent decades, learning has been assumed rather than investigated in clinical research and practice [1]. With the return of the cognitive revolution, modern associative learning theory has expanded the behaviourist model to suggest more powerful mechanisms. In particular, changes in behaviour are now explained in terms of internal processes by which the mental representation of one event (i.e., a stimulus or response) becomes linked to another in memory through experience. Interestingly, such associative processes have been observed in the development and regulation of habitual eating, including our likes and dislikes, choosing foods most appropriate to our current motivational state, and controlling how much is eaten [2,3,4]. Regarding disordered eating behaviour, modern associative learning theory also enables a deeper understanding of the psychological processes of abnormal behaviour, as well as promoting clinically effective empirically supported psychological interventions (e.g., see [5,6]). In this sense, associative processes are now considered among the best candidates to produce reliable findings for translation into advanced research and, consequently, better treatments for eating disorders (EDs). Likewise, it is reasonable to expect that, in the coming years, there will be a rise in the number of papers focused on EDs and associative learning mechanisms.

Moreover, associative learning theory must play an important role in the current paradigm shifts within the psychopathology of EDs. These paradigm shifts include the Research Domain Criteria (RDoC) initiative, precision medicine, experimental psychopathology and cognitive-behavioural psychopharmacology. Herein is an overview of these paradigm shifts in EDs (particularly anorexia nervosa) and the potential benefits of considering associative learning theory, followed by a brief explanation of how the studies in this Special Issue could advance the field.

## 2. Current Paradigm Shifts in Eating Disorders and Associative Learning

*The RDoC initiative*. In the *Diagnostic and Statistical Manual of Mental Disorders, Fifth Edition* ([DSM-5]; the American Psychiatric Association [APA], 2013) and the *International Classification of Diseases* (World Health Organization, 2019; https://icd.who.int/browse11/l-m/en; accessed on 1 November 2022), EDs such as anorexia nervosa and bulimia nervosa are characterised by symptoms (e.g., excessive preoccupation with body weight or shape). Nevertheless, these symptom-based classifications suffer from important flaws such as disorder heterogeneity, overlapping diagnostic criteria and cross-over from one diagnosis to another [7]. Additionally, high rates of co-occurrence among disease categories and substantial variability in symptom presentation within the same diagnosis are observed in EDs (see [8] for a review). This complicates the advancement of their treatment and treatment outcome predictions [9]. In response to these limitations, the US National Institute of Mental Health (NIMH, 2019; https://www.nimh.nih.gov/research/research-funded-by-nimh/rdoc/index.shtml; accessed on 1 November 2022) launched the RDoC initiative. RDoC is a research framework for investigating mental disorders that considers psychopathology in the context of the major domains and constructs of basic human neurobehavioural function, rather than within established diagnostic categories. Interestingly, recent studies highlight main dysfunctions of EDs according to the eating- and weight-related RDoC domains in patients [9,10], with a growing interest in the use of the RDoC approach (e.g., [11]). Moreover, they are using learning theory to explain the possible interplay/interface of RDoC constructs over time in EDs. An example is provided by Schaefer & Steinglass [12], whereby they regard reward prediction error as an area of interest to further understand the mechanisms of EDs within the NIMH-designated RDoC constructs of positive valence systems.

*Precision medicine and precision clinical psychology*. A benefit of mechanism-oriented approaches is their apparent ability to identify individuals at risk, improve diagnostic accuracy and offer personalised (patient-tailored) treatment. Such disease- and patient-specific therapies are now generally called precision medicine and precision clinical psychology, or precision psychiatry in psychopathology [13]. The development of such personalised intervention approaches for mental disorders mainly relies on the identification of specific disease markers, endophenotypes and biosignatures that provide objective measures of dysfunction. For instance, in the case of anorexia nervosa, the markers under investigation are largely focused on genetic/genomic factors and have thus far yielded modest results [14]. However, it has been suggested that our understanding of their genetics could be accelerated by investigating gene–environment interactions [15]. An excellent opportunity is provided by the associative learning perspective, in which biological vulnerabilities may be mediated through genetic or neural contributions to promote pathological fear learning (i.e., affecting the speed and strength of fear response acquisition). For example, altered dopaminergic neurotransmission and deficits in learning have been linked in anorexia nervosa [16]. Therefore, elucidating how associative learning processes are altered by genetic, epigenetic, neural or behavioural variables in EDs may improve diagnostic and prognostic assessments and offer new targets for behavioural and pharmacological interventions.

*Experimental psychopathology*. To test predisposing and etiological factors in EDs, experimental psychopathology employs validated models and laboratory-based methods, primarily with preclinical healthy animal and human participants (e.g., analogue samples) [17]. Using this approach, the potential psychopathological process is often induced directly in the laboratory in mild but prototypical forms [18]. These preclinical models are not only crucial tools for developing markers of diagnosis, drugs for therapy and the identification of clinical targets; they are also expected to serve as the cornerstone of comparative psychiatry and clinical psychology, translational research and precision medicine in EDs [19]. Thus, new learning paradigms are being applied in preclinical animal studies, demonstrating, e.g., the impact of dysfunctional thoughts on the occurrence of food restriction in rats. In particular, in an animal model, it was reported that the acceptance of a sugary solution decreases without the animals ever having directly experienced unpleasant physical events, but by means of “disgusting” mental representations of foods (e.g., [20]). Novel applications of this new generation of learning paradigms is expected to increase the face, diagnostic, predictive and construct validity of current animal models of EDs.

*Cognitive-behavioural psychopharmacology*. To improve treatment outcomes, traditional pharmacotherapy has often been combined with psychotherapy. Unfortunately, both types of interventions have developed as separate, non-complementary and sometimes competitive entities. Indeed, incompatibility and adverse interactions between pharmacotherapy and psychotherapy have been observed to have disruptive effects on one-another. For instance, the use of traditional antidepressants and anxiolytics appears to cause attenuated extinction learning during exposure therapy [21]. Interestingly, a new paradigm in clinical psychopharmacology now embraces biopsychosocial orientation in the treatment of mental illness: cognitive-behavioural psychopharmacology [22]. In this paradigm, for instance, cognition-enhancing medication is prescribed to improve new associations during psychological therapy. An example is D-cycloserine, which appears as an adjunct to exposure-based psychotherapy to accelerate the extinction learning processes. Interestingly, preliminary data suggest an enhancement in the effects of exposure therapy in anorexia nervosa and increased weight gain in randomised controlled trials [23]. Nevertheless, research in this new field of behavioural psychopharmacology is still in its infancy, but much is expected from this coordinated integrative perspective guided by associative mechanisms.

## 3. The Contents of This Special Issue

This Special Issue contains interesting contributions that will shed light on the associative learning processes underlying anorexia nervosa while considering associative learning theory as an interdisciplinary, transdiagnostic and translational platform that includes data from animals, healthy individuals and patients. To this end, this issue already covers several topics; these range from clinical open intervention studies, in which patients with anorexia nervosa are treated with a new form of exposure based on inhibitory learning, to experimental studies into avoidance behaviours, such as those conducted by Anita Jansen and colleagues. Additionally, this issue includes review articles that contribute to explaining food restriction in anorexia nervosa; from the associative account of learning, by Peter Wilhelm, Claus Vögele and Simone Munsch, to the relationship between habitual learning and dietary restraint, by Monica Leila Portela and colleagues. Nevertheless, we expect to encounter more studies that aim to successfully improve clinical research and practice for anorexia nervosa. It seems clear that the application of associative learning in EDs constitutes a driving force for innovation in the upcoming and ongoing paradigm shifts. Moreover, the emergence of new associative experiments is likely to alter not only our perception of EDs, but also their therapy in the coming years. For clinicians and patients, the associative learning framework is sure to lead to major changes in the treatment of EDs such as anorexia nervosa, and I hope that the current Special Issue will stimulate further clinical research.
